# The Knowledge and Attitudes of Primary Care and the Barriers to Early Detection and Diagnosis of Alzheimer’s Disease

**DOI:** 10.3390/medicina58070906

**Published:** 2022-07-07

**Authors:** Donna de Levante Raphael

**Affiliations:** National Memory Screening Department, The Alzheimer’s Foundation of America, New York, NY 10001, USA; draphael@alzfdn.org; Tel.: +1-(917)-688-8518

**Keywords:** Alzheimer’s disease, primary care physicians, dementia, knowledge and attitude, early diagnosis and management, barriers to diagnosis

## Abstract

Primary care physicians play a vital role in the clinical care of their patients, early identification of dementia, and disease advocacy. It is essential to assess the knowledge and attitudes of physicians in the diagnosis of Alzheimer’s disease and other dementias. In primary care, the diagnosis of Alzheimer’s disease is often missed or delayed. With the increased prevalence of Alzheimer’s disease and the growing impact of dementia on health care resources, early detection by primary care physicians (PCP) is essential. Thus, their knowledge and attitudes about early detection and diagnosis are crucial. To examine the knowledge and attitudes of primary care physicians regarding early detection and diagnosis of Alzheimer’s disease and how barriers may contribute to missed and delayed detection and diagnosis. An interpretive scope review was used to synthesize and analyze a body of literature published over the past decade. The study population are physicians in the United States. The current health systems experience challenges in providing early, safe, accurate, and comprehensive Alzheimer’s diagnosis and care by a primary care physician trained or knowledgeable in diagnosing the various forms of dementia. This article identifies several interrelated obstacles to early detection and diagnosis in primary dementia care, including gaps in knowledge, attitudes, skills, and resources for person with dementia (PWD)/caregivers and their primary care providers and systematic and structural barriers that negatively impact dementia care. Research shows that Alzheimer’s disease has gone underdiagnosed and undertreated. Delays in detection, diagnosis, and resource utilization may have social and clinical implications for individuals affected by Alzheimer’s disease and their families, including challenges in obtaining an accurate diagnosis. Until the issues of missed and delayed Alzheimer’s screening become more compelling, efforts to promote early detection and diagnosis should focus on the education of physicians and removing the barriers to diagnosis.

## 1. Introduction

This study aims to examine how the knowledge and attitudes of primary care physicians (PCP) contribute to the barriers to early detection and diagnosis of Alzheimer’s disease (AD) using an interpretive scoping review to synthesize and analyze an extensive body of literature on this topic. Alzheimer’s disease is a significant and growing public health issue in the United States for which early detection and diagnosis are essential. An estimated 6.5 million Americans live with AD right now. The projected number of people is estimated to reach 12.7 million by 2050 [1]. America’s health care will be challenged in both training and size. Currently, Alzheimer’s diagnosis in the primary care setting has been dependent mainly on clinical suspicion based on the patient’s or caregiver’s concerns rather than the use of assessment tools and is often prone to missed or delayed diagnoses. Early accurate detection and diagnosis are consistent with high-quality care and offer several direct benefits to individuals with AD. For example, treating reversible causes of dementia, implementing interventions to slow the progression of the disease, and commencing advanced planning while the patient is still competent. Other examples may be receiving access to education, participating in clinical trial options, engaging family and caregivers with support resources, and potentially delaying institutionalization [2]. Alzheimer’s disease has a wide range of adverse consequences, including functional limitations affecting the routine performance of daily living and self-care activities, complications due to other co-existing medical conditions, increased healthcare services utilization, and increased caregiver burden. Major contributory factors to missed and delayed detection and diagnosis often include issues with knowledge deficits and attitudes of healthcare professionals and patient-care provider communication [3]. Over the past 20 years, there has been a global commitment to seeking a more active approach to PCPs for older adults with dementia, with PCPs again at the center of attention. Experts have repeatedly acknowledged the crucial role of PCP in delivering an early diagnosis, responsive treatment, overall care management, and provision of support services to older adults with dementia and their families. The overall goal of this article was to identify the barriers to providing optimal primary Alzheimer’s care and reduce missed and delayed detection and diagnosis. Until the issue of missed and delayed Alzheimer’s screening is addressed, efforts to enhance the use of screening tools by primary care physicians and remove the barriers to their use are essential to promoting early detection and diagnosis.

Due to the increased prevalence of AD and other dementias, the United States healthcare systems are shifting the care of these patients to primary care [3]. Many patients and caregivers view their primary care physician (PCP) as their key point person for managing their care. Thus, PCPs are a significant stakeholder in our care system for people with Alzheimer**’**s and other dementias. The critical role of the PCP includes early screening, identification, and diagnosis of dementia; outcome/course of the disease; and support services available in the community. However, the signs and symptoms of AD and other dementias often are insidious and difficult to diagnose in the early stages of the onset of dementia.

## 2. Materials and Methods

The methodology used for this review was an interpretative scoping review based on the framework developed by Arksey and O’Malley and the more recent work of Davis and colleagues. This framework was used to guide the review process. This methodology systematically examines, synthesizes, and analyzes an extensive body of relevant literature. The extensive nature of this type of review offered a mechanism to thoroughly and systematically map various forms of the existing evidence, including a range of primary research and non-research sources. An interpretive approach established an in-depth scope and interpretive analysis of the findings that inform future research, practice, and policy.

An electronic search of nine databases was conducted to secure relevant information on geriatric and gerontology topics: MEDLINE, EbscoHost, ProQuest, Google Scholar, PsycARTICLES, PsycINFO, CINAHL, PubMed, and the National Institute on Aging. The search strategy used to incorporate the selected 22 articles was the Boolean method. This method allowed the combination of modifiers and operators with keywords, such as Cognitive impairment, dementia, Alzheimer’s disease, dementia, training, screening, barriers, assessment, detection, diagnosis, or educational interventions. To extract the relevant literature for this review, the following key search terms and combinations of search terms to search for the related literature resulted in: primary care physician, primary care, primary health care, physician, family doctor, family physician, general practitioner, AND dementia, Alzheimer’s Disease, or cognitive disorders, or cognition AND Alzheimer’s disease diagnosis, early diagnosis, early detection unmet needs, support, knowledge, dementia training, and barriers to diagnosis. Both the PICO question and the framework were used to guide the search and find relevant keywords for the literature review.

## 3. Results

**Figure 1:**
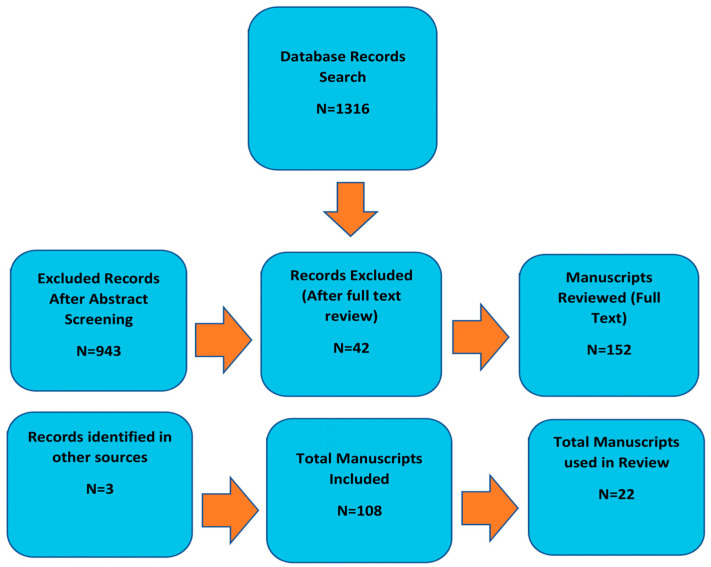
Flow Chart of Manuscript Identification and Selection.

Over the past two decades, there has been an extensive list of papers on the clinical practice, best practice recommendations, and evidence of actual practice guidelines for diagnosing and managing the care of people with AD or another dementia. Despite the many forms of care systems, they are consistent with the recommendations that patients experience changes in their cognition and should first seek care with their primary care physician (PCP). The chart Figure 1 is the Flow Chart of Manuscript Identification and Selection of the extensive list of papers reviewed and those that contributed in part to the practice guidelines for diagnosis and care management of patients with dementia. It is agreed that this PCP should first begin care with the identification of the early signs and symptoms of AD or another dementia. The second step is followed by a multidisciplinary evaluation, a collaborative care plan with the input of other support team members, and ongoing follow-up monitoring and management.

A substantial number of PCP throughout several studies acknowledged that though there are challenges in primary care, diagnosis can be conducted by PCP in the form of clinical evaluations, brief cognitive testing, lab tests, and structural imaging, if necessary. However, this agreement on the clinical practice of dementia research has consistently shown a lack of consensus between agreed-upon best practice recommendations and actual acts related to dementia diagnosis and management [3,4,5].

According to several studies, general concerns of PCPs or general practitioners (GP) show that the early stages of AD and other dementias remain under-detected, under-diagnosed, under-disclosed, under-treated, and mismanaged [6,7,8]. Several study surveys revealed that the PCPs agreed with the enablers of early AD or dementia recognition, such as planning for the future and arranging care and support. At the same time, some respondents perceived barriers to early diagnosis, such as time limits to carrying out diagnosis. Evidence shows that AD and other dementias are mainly diagnosed at the middle to late stages of the disease and not sufficiently disclosed or followed up with a timely, comprehensive approach [4,8]. Dementia diagnosis delays often occur even though suspicion of the disorder is brought about by family members or the presence of negative cognitive screen results [7,9].

In a few recent large-scale international surveys, it was confirmed that physicians and the general public had significant difficulties in recognizing and acknowledging early dementia symptoms. There were also significant delays in seeking help and providing a diagnosis. According to these studies, from the initial presentation of dementia symptoms to the diagnosis of dementia, it often took months to years. Delays occurred from the initial recognition of symptoms by family to physician consultations [5,7,9]. Throughout the studies, family caregivers generally waited approximately two to three years before reporting the presentation of symptoms to the PCP [8]. Though there were high rates of referrals of suspected presentations of dementia from PCP and other specialists, referrals did not necessarily lead to a proper diagnostic investigation. Though dementia was detected and documented in patient medical files, PCP failed to disclose the diagnosis and did not follow up with their patients [9,10]. According to the studies many PCP reluctantly shared the diagnosis of dementia.

Failure to disclose a diagnosis as significant as dementia may be unethical. There are many benefits to an early dementia diagnosis disclosure. A review paper summarizing studies reported an estimated 50 percent of physicians standardized withholding dementia diagnosis from their patients [8]. In comparison, 71 percent of the survey respondents without cognitive impairment indicated a solid desire to be informed of a dementia diagnosis [9,11,12]. The surveys also revealed that patients felt that it was critical to be informed of their diagnosis so they could access information, plan, and participate in treatment options [11,12,13].

### 3.1. Main Challenges Encountered

Though there are structured processes for other health conditions, there are none for cognitive diseases [8]. There are no specific standardized tests or guidelines established for cognitive assessments. The cognitive assessments and diagnosis approach has been reactive when patients raise issues or self-report their cognitive experiences or challenges. This approach can make cognitive screenings challenging in a clinical setting. Some of these challenges may include:

- The PCP does not have special dementia training for cognitive screenings;
- Screenings cause a delay in the clinic’s workflow;
- Time limitations, fear of giving a diagnosis;
- Lack of cultural competency in the patient’s understanding of the cognitive changes;
- Not knowing how to start the conversation;
- Alzheimer’s disease stigma;
- Fear of harm to the older patient by conducting a cognitive assessment that may lead to depression or anxiety;
- Discrimination.

With these challenges and barriers, many PCPs remain hesitant to initiate the concerns of cognitive testing with their older patients [14]. Providers often wait for the older adult or family member to initiate the conversation about their problems.

Physicians are in an ideal position to observe potential signs of cognitive decline and ask pertinent questions. As the provider to the individual, they may have long-established relationships with the individual and their family. When the patient is concerned about any changes regarding their memory functions, they would most likely take the concern to their providers [14]. However, older adults will rarely initiate the conversation about their cognitive challenges for various reasons. Some reasons may include:

- Fear;
- Cultural perceptions of the disease;
- Stigma;
- Past experiences;
- Not believing there is a benefit to knowing about the disease;
- Why bother? Dementia is incurable.

### 3.2. Barriers to Diagnosis—PCP Attitudes and Perceptions

Throughout this review, the literature disclosed many interrelated obstacles and challenges, such as the complex nature of AD and other dementia disorders and significant gaps in knowledge, skills, resources, and attitudes of PCP, patients, and caregivers. Additional significant barriers were reported as the systematic and structural barriers which negatively affect dementia care. There is significant evidence that PCP experience difficulty recognizing early AD or other dementia symptoms and overlook the importance [5,8,15]. For example, many PCP conveyed low confidence in producing a dementia diagnosis, especially in the early stages of the disease. They often felt their training was insufficient to prepare them to accurately and confidently screen or give diagnoses [15,16,17] and preferred their patients to participate in a specialist consultation [16]. The studies also showed that many PCP viewed dementia diagnosis and management to be more complex than other chronic conditions.

Many PCPs and other medical providers are unaware of Medicare’s mandatory annual cognitive assessment for older adults aged 65 and older. There is also difficulty managing communication and management skills. Many PCP also admitted that they felt uninformed about the next steps after diagnosis and the available support services for patients and caregivers [12,15,18].

A significant body of research has shown that PCP dementia diagnosis and management practices may be influenced by their beliefs and attitudes. The literature also highlighted that those PCPs who had negative attitudes and perceptions might threaten their commitment to early diagnosis and disease management. A few of these perceptions concern the lack of real therapeutic benefits of early diagnosis and disclosure leading to depression and anxiety in a person with dementia (PWD) and their caregivers. Other perceptions are concerned with the harmful effects of the various forms of stigma experienced immediately upon diagnosis. Throughout the disease, low priority is given to dementia symptoms instead of physical health issues and the belief that care for the diagnosed person would increase the strains of the already strained health care system [8,13,17]. The question of insufficient time was a significant issue as it constrains the ability of the PCP to provide optimal care to the patients and caregivers. Insufficient time was also the primary and most significant barrier to optimal dementia care, according to PCPs [17]. Another issue was the inadequate payment models and reimbursement structures that did not accurately reflect the time needed to care for the needs of the elderly patient, especially those with AD or other dementia [17].

### 3.3. Attitudes

Though there was wide understanding, agreement, and confirmation of the benefits of early detection and diagnosis of AD and other dementia, PCP attitudes toward AD and other dementia, including administering cognitive assessments, disclosing diagnosis and guiding and referring patients to specialists, and caregivers to community based organizations, revealed that they felt strongly that their level of confidence in their ability to perform the aforementioned care was significantly associated with their lack of dementia specific training and knowledge that would allow them to improve their overall care and support to dementia patients.

PCPs’ attitudes reflected that it was important to assess older adults for cognitive impairment from the age of 65 years. It also reflected their understanding that early detection and diagnosis of cognitive decline had benefits to the individual to include social, financial, medical, and planning. In several studies, PCPs felt that patients were too ill to proceed with cognitive testing.

Other attitudes reflected that there was a higher barrier in the United States compared to other countries utilizing the PET testing regularly. In the United States the barrier was that testing was significantly lower than other countries due to the cost and challenges around the reimbursement of the PET imaging process. Attitudes toward the routine use of objective tests during the Medicare Annual Wellness Visit (AWV) are more common in the United States. The required AWV implemented in 2010 required direct observation of cognitive function, concerns, and symptoms that should initially prompt cognitive testing.

### 3.4. Barriers to Diagnosis—PCP Knowledge

Meeting the educational needs of PCPs has become crucial to diagnosing and management of older adult patients. Various knowledge-based interventions have been developed and utilized with varied success. Provider-related barriers included lack of training and confidence in knowledge. According to seven studies, approximately 10–63% of respondents lacked knowledge and training [19]. Lack of confidence or comfort due to knowledge and skill deficits was reported by 23–66% of respondents in four studies [12]. Many PCPs often find themselves having to discuss dementia diagnosis despite their limited knowledge of symptoms, causes, treatment options, and other diseases or reversible dementias that present with cognitive symptoms [12]. A recent study with 343 PCPs working in hospice settings showed that PCPs tended to give inaccurate information about their prognosis, and the errors were highly optimistic instead of very pessimistic [12]. This behavior extended the patient/physician relationship and delayed the diagnosis. Gaps in knowledge and skills and an understanding of dementia can stigmatize people affected by dementia. They can also result in barriers to health care access, diagnosis, and quality of care. Though some medical education programs offer education on dementia, very few offer in-depth focus on cognitive health and aging [18].

Education content of AD or other dementias and geriatric education has not been standardized and varies significantly across the board. Education in family medicine programs ranges from optional to one lecture or a tour of clinical rotation in geriatrics [18]. This content is not significant enough to be knowledgeable enough to deliver appropriate care [15,17,18]. The studies also called attention to the situation that very few family medicine students receive or pursue opportunities in caring for the older adult population [15,17,18]. Furthermore, the older adult population with dementia in the United States is diverse, and family medicine students will need to provide quality care. To deliver quality care, medical students require clinical training and education, including dementia education that recognizes the unique needs of the various socio-cultural groups that their patients identify with and those in different geographical settings [15,17,18].

Given the United States’ aging population, there is a critical gap in the requirements for improved education and training on dementia in family medicine training programs to improve early dementia detection, diagnosis, treatment, management, and quality of care [20]. The health care field requires more education on risk reduction strategies; proactive management vital to addressing nihilistic attitudes, with the belief that to provide a patient with a dementia diagnosis that has no cure is providing them with a diagnosis that was not actionable in a clinical manner [20]; stigmatic beliefs; helplessness and strain of the healthcare system; and the widespread under-diagnosis of dementia. Furthermore, it is crucial to be educated and abreast of non-pharmacologic interventions that can support brain health promotion [13]. Though there is no known cure for AD or other dementia, education on risk factors that are modifiable is vital because it can delay the onset or slow the progression of the disease [13].

Consequently, when dementia education is prioritized to reduce stigma and improve brain health promotion, early diagnosis, and quality of care for people with AD or another dementia, patients are satisfied, and patient outcomes improve. In working to elevate physician education on AD and other dementia, an essential source of information is the Alzheimer’s Foundation of America (AFA). They offer educational programs and materials for both the communities and professionals who work with those with dementia, persons with dementia, and their caregivers and provide resources on memory screening and diagnosis. They also run the national memory screening program, which allows individuals concerned with memory loss and other cognitive changes to be screened. Though not a diagnosis of any illness, this screening offers the individual the opportunity to obtain a baseline of their thinking skills and then return to follow up at a later date. When there is an apparent concern, the AFA recommends the screened individual to follow up with their PCP. The Alzheimer’s Foundation of America also provides the opportunity for healthcare providers to refer people with cognitive impairment, diagnosed or undiagnosed, to receive accurate information and support on AD and other dementias.

Throughout the various studies there was no indication that health insurance played a role as a barrier to PCPs conducting cognitive assessments. The Medicare Wellness Visit (AWV) plan covers older adults ages 65 years and older. The cognitive assessment is part of a routine visit and includes a brief cognitive test. Medicare covers a separate visit to conduct a more detailed cognitive assessment and development of a thorough care plan.

## 4. Discussion

### 4.1. Early Dementia Diagnosis

Most efforts in the United States to improve primary dementia care have been either isolated or limited in scope, usually addressing any minor subset of barriers with a modest intensity and limited coordination [19]. Many experts believe that achieving meaningful and sustained improvements in dementia diagnosis and disease management and care should ideally be developed and mandated by active and specific national dementia strategies [19]. The evidence reviewed suggests that timely diagnosis and quality care of people with AD or other dementia is more an exception than a rule in many parts of the United States. Research has steadily shown that dementia diagnosis commonly occurs in the middle to later stages of the disease [21]. Diagnosis often occurs at the time of crisis [21].

### 4.2. Barriers to Diagnosis—Professional and Public Education

Dementia education is key to positively affecting professional providers’ and the public’s help-seeking behaviors. Evidence suggests low dementia awareness of the early signs and symptoms of AD or another dementia. There is evidence that delayed PCP responses may be due to a limited understanding of disease experience, attitudes associated with nihilism, stigma, ageism, and deficits in diagnosis disclosure, communication, and disease management skills.

Currently, most educational efforts to enhance PCP practice focus on improving their formal knowledge of dementia, such as pathophysiology and pharmacology. Primary care physicians believe that educational interventions should have a broader scope that addresses the gaps in attitudes, behaviors, knowledge, and skills. The term “knowledge” should include disease recognition, conceptual framework, and therapeutic interventions and their perspectives in learning and support requirements to provide AD and other dementia care. Relevant topics to explore more thoroughly and that would be relevant to PCP and other medical professionals are:

(a) Professional and public expectations of PCP roles and responsibilities;
(b) Consistent, standardized, and effective educational tools and training strategies;
(c) Integrated models of dementia care (e.g., feasibility and long-term care cost-effectiveness;
(d) Dementia care collaborations between PCPs and specialists;
(e) Barriers and incentives to PCP participation in multidisciplinary dementia care and delivery systems.

Finally, the generation of new knowledge revised cognitive assessment tools that consider the diverse older adult population differences in information, education, experiences, culture, language, beliefs, and attitudes. There is also a critical need to effectively transfer knowledge gained and convert the evidence into tangible and substantial practice and interventions.

Without forward and consistent movement on recommendations, AD and other dementias may be the main problem, causing more under-detection, under-diagnosis, and misdiagnosis that escalates caregiver burden, additional illnesses, and economic issues. Therefore, medical education regarding AD and other dementias should evolve from a mainly disease-focused emphasis to a broader view that dementia is a complex, chronic, and progressive disorder that can be responsive to early, comprehensive, and personalized treatment and management plans. These plans should focus on the support of PCPs in their practice and plans and would require a shift in acquiring new and diverse skills in medical training and practice, including in wider society [22]. Consistent medical training should be a part of awareness-raising and educational interventions to reexamine dementia more accurately and increase the public’s understanding of it [22].

## 5. Implications to Advance Physician Practice

These studies represented the challenges primary care physicians must overcome to improve early detection and diagnosis of cognitive impairment related to AD and other dementias. Moreover, they reveal the need to train physicians, medical students, and other medical staff to screen the cognitive functions of patients, especially those 65 years and older. Furthermore, the implementation of either national or statewide educational programs that help educate the medical staff will contribute to increasing their clinical skills and equip them to accurately screen patients’ cognitive functioning. Hence, identifying cognitive changes leading to AD or other dementias. According to Islam et al. (2020), the training of medical staff in primary care needs to be ongoing from medical school through practice to ensure long-term sustainability.

## 6. Limitations

One of the primary limitations of this review was that the studies were only targeted to primary care physicians and were not inclusive of other healthcare professionals. Furthermore, another limitation is that many of the studies focused on primary care physicians who belonged to medical centers. Furthermore, another constraint worth mentioning is the integrity of the participants. Moreover, being able to identify studies that represent the diverse population of physicians in the United States is difficult.

## 7. Recommendations

Cognitive impairment requires the attention of the healthcare community because it is a medical condition. Cognitive impairment assessment has been included in the Medicare Annual Wellness Visit (AWV) since it was implemented in the Affordable Care Act in 2010. However, in primary care, cognitive impairment, AD, and other dementias have been under-diagnosed. According to the Alzheimer’s Association Report (2019), approximately 50 percent of patients have their cognitive functioning routinely assessed by primary care physicians. This literature review was used to understand and improve PCPs cognitive screening accuracy, knowledge, and confidence.

## 8. Conclusions

This review was conducted to determine whether educational intervention in a primary care setting would improve cognitive screening accuracy and rates. The review concluded that educational intervention was needed to train PCPs on the proper use of cognitive assessment tools and to initiate the conversations about memory loss and cognitive functioning in patients aged 65 years and over. This is essential to reducing the dementia detection and diagnosis gap. It is necessary for organizations to have a culture that promotes learning. Strong leadership receptive to change may push the system towards mandatory training for dementia, but PCPs need to follow the set guidelines. More work is needed to overcome the barriers associated with the implementation of interventions in order to increase feasibility and effectiveness. Further research can contribute to a better understanding of the disease and the experience of U.S. PCPs. Consistent forward-moving strategies and actions are needed to change attitudes and decrease knowledge deficits as the numbers of older adults with AD and other dementias increase.

## Data Availability

Not applicable.
